# A Novel Naturally Occurring Tandem Promoter in Modified Vaccinia Virus Ankara Drives Very Early Gene Expression and Potent Immune Responses

**DOI:** 10.1371/journal.pone.0073511

**Published:** 2013-08-12

**Authors:** Sonia T. Wennier, Kay Brinkmann, Charlotte Steinhäußer, Nicole Mayländer, Claudia Mnich, Ursula Wielert, Ulrike Dirmeier, Jürgen Hausmann, Paul Chaplin, Robin Steigerwald

**Affiliations:** Infectious Disease Division, Bavarian Nordic GmbH, Martinsried, Germany; The Scripps Research Institute and Sorrento Therapeutics, Inc., United States of America

## Abstract

Modified vaccinia virus Ankara (MVA) has been shown to be suitable for the generation of experimental vaccines against cancer and infectious diseases, eliciting strong humoral and cellular immune responses. In viral vectored vaccines, strong recombinant antigen expression and timing of expression influence the quantity and quality of the immune response. Screening of synthetic and native poxvirus promoters for strong protein expression *in vitro* and potent immune responses *in vivo* led to the identification of the MVA13.5L promoter, a unique and novel naturally occurring tandem promoter in MVA composed of two 44 nucleotide long repeated motifs, each containing an early promoter element. The MVA13.5L gene is highly conserved across orthopoxviruses, yet its function is unknown. The unique structure of its promoter is not found for any other gene in the MVA genome and is also conserved in other orthopoxviruses. Comparison of the MVA13.5L promoter activity with synthetic poxviral promoters revealed that the MVA13.5L promoter produced higher levels of protein early during infection in HeLa cells and particularly in MDBK cells, a cell line in which MVA replication stops at an early stage before the expression of late genes. Finally, a recombinant antigen expressed under the control of this novel promoter induced high antibody titers and increased CD8 T cell responses in homologous prime-boost immunization compared to commonly used promoters. In particular, the recombinant antigen specific CD8 T cell responses dominated over the immunodominant B8R vector-specific responses after three vaccinations and even more during the memory phase. These results have identified the native MVA13.5L promoter as a new potent promoter for use in MVA vectored preventive and therapeutic vaccines.

## Introduction

Replication-competent poxvirus vectors have been employed as vaccine vectors since the 1980s to induce immune responses against encoded foreign genes [1,2]. However, vaccines based on replication competent poxviruses, such as vaccinia virus (VACV) pose safety issues, particularly in young children and immunocompromised individuals [3,4]. To enhance the safety of VACV, highly attenuated, replication-restricted viral vectors such as the modified vaccinia virus Ankara (MVA) and the VACV Copenhagen (VACV-COP)-derived NYVAC strains have been developed (reviewed in 5).

MVA was derived from chorioallantois vaccinia Ankara (CVA), a Turkish smallpox vaccine strain, by serial passaging (> 570 passages) in primary chicken embryo fibroblast (CEF) cells [6,7]. During passaging, the host range of the virus was severely restricted and, as a consequence, MVA is unable to productively infect many mammalian cells [8–10]. Host range restriction of this virus in cell culture is partially governed by the six major deletions and is to a large extent based on additional mutations among the multitude of genes with altered amino acid sequence compared to the parental CVA virus [11,12]. As a third-generation smallpox vaccine, MVA has shown an excellent safety profile and immunogenicity in the clinic [13–15]. In addition, MVA is well tolerated and immunogenic when administered to immunocompromised patients infected with human immunodeficiency virus (HIV), highlighting its potential as a safe vector for the development of vaccine and gene therapy candidates [16].

The expression of foreign genes from poxvirus vectors is achieved by the use of native or synthetic poxvirus promoters, which are recognized by the viral transcription machinery. It has been shown that the levels of recombinant antigen expressed from MVA infected cells *in vitro* correlates with the magnitude of the immune responses in mice [17,18]. In addition, temporal regulation of antigen expression also plays a role particularly in the induction of CD8 T cell responses against VACV proteins during infection. Poxviruses control their gene expression at the level of transcription through a cascade-like mechanism involving three major classes of genes; early, intermediate, and late with the latter two classes being expressed after genome replication (reviewed in 19] [18). VACV genes expressed early during infection tend to be preferentially recognized by CD8 T cell responses, while VACV genes expressed at intermediate and/or late times post infection tend to be preferential targets for CD4 T cell and antibody responses [20–22]. In MVA, very early expression of a recombinant antigen can also enhance the specific CD8 T cell responses against the recombinant protein upon repeated immunizations [23].

Promoters with both early and late activity are commonly used to direct the expression of foreign antigens in poxvirus vectors to ensure that adequate expression levels are present at the appropriate time for induction of strong immune responses. Some of these promoters are native poxvirus promoters that drive the expression of a viral protein, such as p7.5k [24] and the modified promoter H5 (H5m) [25], while other promoters such as the short synthetic early/late promoter, PrS [26], were designed *in silico* based on early [27] and late [28] consensus motifs observed in native poxvirus promoters. In addition, synthetic promoters employing multiple early elements and the late A-type inclusion body (ATI) promoter have also been characterized [17,23,29]. In particular, the promoter pHyb has been shown to drive the expression of antigens earlier during infection when compared to the PrS and p7.5k promoters and also to induce stronger CD8 T cell responses after repeat vaccinations [23]. In that report [23], the pHyb was shown to function as an immediate-early promoter or E1.1 (defined in [30,31]) with regard to timing of expression. However, effects of clustering multiple late promoter elements with early promoter elements have not yet been described.

Still other reports have focused on the use of endogenous MVA promoters *in situ* (i.e. at their natural site in the MVA genome) by the deletion of the downstream genes and the subsequent insertion of a transgene at the deletion site [32]. This method provides the advantage of using the promoter within its natural context in the MVA genome and avoids the need to determine the particular promoter sequences driving the expression of the gene. However, by deleting these MVA genes, new viral vectors are essentially developed with unknown characteristics in terms of their replication capacity, and their ability to stimulate strong immune responses.

Two recent reports have provided an analysis of the VACV transcriptome, which include information about the temporal expression of all viral genes during VACV infection [30,31]. In addition, the 5’ and 3’ ends of the VACV early mRNAs have also been characterized along with potential transcriptional start sites and putative promoter sequences [30,33]. This wealth of information can facilitate and simplify the identification of potential promoter sequences from early MVA genes. Novel native promoter sequences from genes expressed at high levels early during infection can be identified, cloned and used for the expression of transgenes inserted at intergenic regions, thus avoiding the alteration of genes from the MVA vector.

In this report, we directly compare the kinetics, strength, and immune responses directed by previously characterized promoters against those driven by novel synthetic and native MVA promoters using a model recombinant antigen in MVA. In particular, we characterized the activity of two novel hybrid promoters, Pr4LS5E and PrS5E, and analyzed the effects of clustering multiple late and early elements into one promoter. In addition, we describe the identification and characterization of a new MVA-derived native tandem promoter, PrMVA13.5-long. This native tandem promoter induced strong immediate-early (or E1.1) expression and drove potent CD8 T cell responses after repeat vaccinations along with strong humoral responses, making it an ideal promoter for use in recombinant MVA-based vaccines or immunotherapies.

## Materials and Methods

### Ethics statement

All animal studies were approved by the local authorities of the Regierung von Oberbayern (Government of Upper Bavaria) and were carried out in accordance with the regulations of the German animal welfare law set forth by this authority (permit number: 55.2-1-54-2531-108-06).

### Cell culture and cell lines

Primary chicken embryo fibroblast cells (CEF) were prepared from 11-day-old embryonated specific pathogen-free (SPF) eggs (Charles River Laboratories, Wilmington, MA, USA) and cultured in virus production - serum free medium (VP-SFM; Life Technologies, Karlsruhe, Germany) supplemented with 4 mM L-glutamine (Life Technologies) and 1% gentamicin (Life Technologies). HeLa cells (ATCC CCL-2) and Madin-Darby bovine kidney (MDBK) cells (ATCC CCL-22) were cultured in Dulbecco’s modified Eagle’s medium (DMEM) with 10% fetal calf serum (FCS) (PAA Laboratories, Cölbe, Germany).

### Generation of recombinant viruses

Recombinant viruses were generated using a bacterial artificial chromosome (BAC) containing the genome of MVA (MVA-BAC) as described in [12]. Briefly, expression cassettes containing the chicken ovalbumin (OVA) gene under the control of known or putative poxvirus promoters were introduced into the MVA-BAC genome at the intergenic region (IGR) between the MVA044L and MVA045L genes (IGR 44/45). All OVA expressing recombinant viruses were generated from the same parental MVA-BAC genome which also contained an enhanced green fluorescent protein (EGFP) expression cassette at the IGR 64/65. The parental MVA virus expressing EGFP (MVA-EGFP) was also reconstituted and served as an empty vector control. The OVA cDNA clone was kindly provided by Christian A. Mohr and U. Koszinowski (Max von Pettenkofer-Institute, Ludwig-Maximilians-Universität München, Munich, Germany). The sequence of the synthetic early/late poxvirus promoter (PrS) [26], was used with the last two nucleotides forming part of the ATG start codon of the OVA open reading frame (ORF). The pHyb promoter has been described in [23]. The sequence of the modified H5 promoter (PrH5m) has been described in [25]. The sequences for the novel hybrid promoters PrS5E and Pr4LS5E and for the MVA native promoters PrMVA13.5 long and short versions are provided in Table 1.

**Table 1:** Sequences of the putative native promoters and novel hybrid promoters.

**Promoter name**	Sequence (5’ to 3’)*
PrMVA13.5-long	**TAAAAATAGAAACTA**TAATCATATAATAGTGTAGGTTGGT AGTATTGCTCTTGTGACTAGAGACTTTAGTTAAGGTACTG**TAAAAATAGAAACTA**TAATCATATAATAGTGTAGGTTGGTAGTA
PrMVA13.5-short	TAAGGTACTG**TAAAAATAGAAACTA** TAATCATATAATAGTGTAGGTTGGTAGTA
PrS5E	*AAAAATTGAAATTTTATTTTTTTTTTTTGGAATATAAATA* **AAAAATTGAAAAACTATTCTAATTTATTGCACGG** TCCGGT**AAAAATTGAAAAACTATTCTAATTTATTGCACGG**TCCGGT**AAAAATTGAAAAACTATTCTAATTTATTGCACGG** TCCGGT**AAAAATTGAAAAACTATTCTAATTTATTGCACGG**TCCGGT **AAAAATTGAAAAACTATTCTAATTTATTGCACGG**
Pr4LS5E	(GTTTTGAATAAAATTTTTTTATAATAAATA)(AATTTTTAATATATAAATA) (TTCTGCATAAATAAAAATATTTTTAGCTTCTAAATA)(TTGATCAATAGTGAAGTTATTGTCAATAAATA) GTTTAAAC*AAAAATTGAAATTTTATTTTTTTTTTTTGGAATATAAATA* **AAAAATTGAAAAACTATTCTAATTTATTGCACGG**TCCGGT**AAAAATTGAAAAACTATTCTAATTTATTGCACGG** TCCGGT**AAAAATTGAAAAACTATTCTAATTTATTGCACGG**TCCGGT **AAAAATTGAAAAACTATTCTAATTTATTGCACGG**TCCGGT**AAAAATTGAAAAACTATTCTAATTTATTGCACGG**

* Sequences in bold correspond to early promoter elements. The PrS promoter sequence is shown in italics. Late promoter elements are separated by parenthesis.

### Rapid amplification of cDNA ends (RACE)

HeLa cells were infected with 10 TCID_50_ (tissue culture infectious doses) per cell with an MVA recombinant virus expressing OVA and cells were collected at 2 and 8 h post infection (p.i.). Cells were homogenised and total RNA was extracted using the RNAeasy extraction kit plus (Qiagen, Hilden, Germany). The residual DNA was removed from the total RNA by DNAse I digest (Roche Applied Science, Mannheim, Germany). A 5’ end RACE assay was performed using the FirstChoice^®^ RLM-RACE Kit (Ambion/Applied Biosystems, Darmstadt, Germany) according to the manufacturer’s protocol using specific oligos (Metabion, Munich, Germany) for amplification of the MVA13.5L gene. The following oligos were used: outer primer (5’-accagttccagattttacacc-3’); inner primer (5’-ccagatgtataagttttagatcc-3’). The amplified cDNAs were sequenced and the 5’ ends indicating the start of transcription were determined by the identification of the linker sequence provided in the kit.

### Reverse transcriptase (RT) – qPCR

HeLa cells were infected with 10 TCID_50_ per cell of the different recombinant MVA viruses expressing OVA or with wild type MVA by cold attachment on ice for 1 h followed by incubation at 37° C. RNA isolation and reverse transcription was performed as described previously [34]. Taqman gene expression master qPCR mix (Applied Biosystems, Darmstadt, Germany) and the following primer and TaqMan probe sets (TibMolBiol, Berlin, Germany) were used for amplification of the OVA transcript: OVA _F (5′-aggattcggagacagtattgaagc-3’), OVA _R (5′-gctgtttgaaagttgataggttcca-3′), and OVA probe (5′- 6-carboxyfluorescein [FAM]- tcgttcagccttgccagtagactttatgct- blackberry quencher [BBQ] -3′). Human β-actin was used as normalizing control and amplified using the Human ACTB (beta actin) VIC dye endogenous control kit (Applied Biosystems) in a multiplex reaction. After uracil DNA glycosylase (UDG) treatment and denaturation for 10 min 95° C, reaction mixtures were subjected to 40 cycles of incubation for 15 s at 95° C and 1 min at 62° C. All reactions were performed on an ABI 7500 real-time PCR cycler (Applied Biosystems). Amplification efficiencies for OVA and actin were determined to be equal. The delta-delta C_T_ method was then used to calculate the fold increase in OVA mRNA expression relative to the undetectable signals in wild type MVA infected cells, which were arbitrarily assigned a C_T_ value of 35, thus representing undetectable results for calculation.

### Flow cytometry staining for OVA protein expression

HeLa cells were infected with 10 TCID_50_ per cell of the indicated MVA recombinants expressing OVA or with MVA-EGFP (empty vector control). Cells were harvested and fixed with 4% paraformaldehyde for 15 min prior to permeabilization with Perm/Wash buffer (BD Biosciences, Heidelberg, Germany). Intracellular staining for the OVA protein was performed using a rabbit anti-OVA antibody (Abcam, Cambridge, UK) diluted 1:1000 for 20 min followed by washing and incubation with an anti-rabbit secondary antibody conjugated to allophycocyanin (APC) (Jackson Immunoresearch, Newmarket, UK) diluted 1:500. All incubations were done at 4° C. Washes and antibody dilutions were done using Perm/Wash Buffer. Samples were analysed, using a BD FACSCalibur flow cytometer (BD Biosciences) and FlowJo software (Tree Star, Ashland, OR, USA).

### ELISA for OVA protein expression

MDBK cells were infected with 10 TCID_50_ of the indicated MVA recombinants expressing OVA or the MVA-EGFP empty vector control. Samples were lysed in PBS containing 0.1% Triton-X100 plus complete protease inhibitor cocktail tablets (Roche Applied Science, Mannheim, Germany). Protein concentration was determined with a bicinchoninic acid (BC) assay protein quantitation kit (Uptima-Interchim, Montluçon, France) and all lysates were diluted to 0.1 µg/µL of total protein. ELISA for the detection of OVA protein was performed using the Serazyme Ovalbumin Kit (Seramun Diagnostica GmbH, Heidesee, Germany). A standard curve was obtained using the Magellan software (Tecan, Salzburg, Austria) and the concentrations of the samples were calculated using the standard curve.

### Immunization experiments

Female C57BL/6 mice aged 6-8 weeks (Janvier, Saint-Berthevin Cedex, France) were immunized via the intraperitoneal (i.p.) route with 1 x 10^8^ TCID_50_ of recombinant MVA viruses expressing OVA, with MVA-EGFP (empty vector) or with PBS (vehicle control) at day 0, 28 and 56. Blood samples were taken at day 7, 35 and 63 for the analysis of OVA- and vector- specific CD8 T cells and at day 21, 49 and 77 for the quantification of OVA- and MVA- specific antibodies. Seven promoter constructs were evaluated in a total of two experiments in which the empty vector, PBS and PrS groups were always included as controls. Thus for these three groups the total number of mice was 8, 9 and 10, respectively. All other groups consisted of 4–5 mice. For memory T cell analysis, spleens were collected 10 weeks after the third immunization.

### MHC class I dextramer staining

Blood samples were collected in PBS containing 2% FCS, 0.1% sodium azide and 2.5 U/mL heparin. Peripheral blood mononuclear cells (PBMCs) were prepared by lysing erythrocytes with red blood cell (RBC) lysing buffer (BD Biosciences) according to the manufacturer’s instructions. PBMCs were immediately stained in a single reaction using anti-CD8α-FITC (BD Biosciences), anti-CD44-PerCP-Cy5.5 (eBiosciences, Frankfurt, Germany) diluted 1:200 and MHC class I dextramers (Immudex, Copenhagen, Denmark) complexed with the H-2K^b^ binding peptides, SIINFEKL (labelled with phycoerythrin [PE]) or TSYKFESV (labelled with APC) for the detection of OVA- and B8R-specific CD8 T cells, respectively. For memory T cell analyses, spleens were collected in RPMI containing 2% FCS. Splenocytes were prepared by mechanical disruption of the spleens through a cell strainer and lysis of RBCs. Splenocyte preparations were stained in a single reaction with both MHC class I dextramers specific for OVA and B8R as well as with anti-CD8α Pacific Blue diluted 1:100, anti-CD44 PerCPCy5.5 diluted 1:200, anti-CD62L-FITC diluted 1:200 and anti-CD127-PE-Cy7 diluted 1:200 (all from BD Biosciences). All stainings were performed in PBS containing 1% FCS for 20 min at 4° C in the dark. Stained cells were washed and analysed on a BD LSR II cytometer (BD Biosciences). Approximately 10000 CD8 T cells were acquired per sample. For memory T cell analyses, 30000 CD8 T cells were acquired per sample.

### Enzyme-linked immunosorbent assay (ELISA) for detection of MVA- or OVA-specific antibodies in mouse sera

Blood samples were collected in serum separating microtubes (BD Biosciences). Serum samples were then serially diluted for the quantification of MVA- or OVA- specific end point antibody titers. For the detection of MVA antibodies, 96-well plates coated with crude extracts of MVA infected CEF cells were used as described in [14]. For the detection of OVA specific antibodies, 96-well plates coated with 0.5 µg/well of chicken OVA protein (Sigma-Aldrich, Munich, Germany) were blocked with PBS containing 0.1% milk and 0.05% Tween-20. After blocking, two-fold serial dilutions of the mouse sera were incubated for 1 h at room temperature (RT). The starting dilution in the assay for negative samples or low titer samples was 1:400. For high titer samples, pre-dilutions of the mouse sera were prepared and higher starting dilutions were used in the assay. Plates were then washed and incubated with a horseradish peroxidase (HRP) conjugated donkey anti-mouse detection antibody (AbD Serotec, Oxford, UK) diluted 1:5 000 for 1 h at RT. All washes and dilutions were done in PBS containing 0.05% Tween-20. Finally, 3,3′,5,5′-Tetramethylbenzidine (TMB; Sigma-Aldrich) was used as the substrate, and the reaction was stopped by adding an equal volume (100µL) of 1 M sulphuric acid (Applichem, Darmstadt, Germany). The optical density (OD) was measured at 450 nm with a Tecan, Sunrise absorbance reader (Tecan, Salzburg, Austria). A regression was obtained from the linear portion of the curves for each sample and used to calculate an end point titer reported as the reciprocal of the dilution giving an OD equal to the cut off value for the assay.

### Statistical analysis

Statistical analysis of data was done by using a one-, a two-way or a two-way repeated-measures analysis of variance (ANOVA) where applicable. Pairwise multiple comparisons between groups were done using the Tukey Test.

## Results

### Construction of novel synthetic hybrid promoters

**Figure 1:**
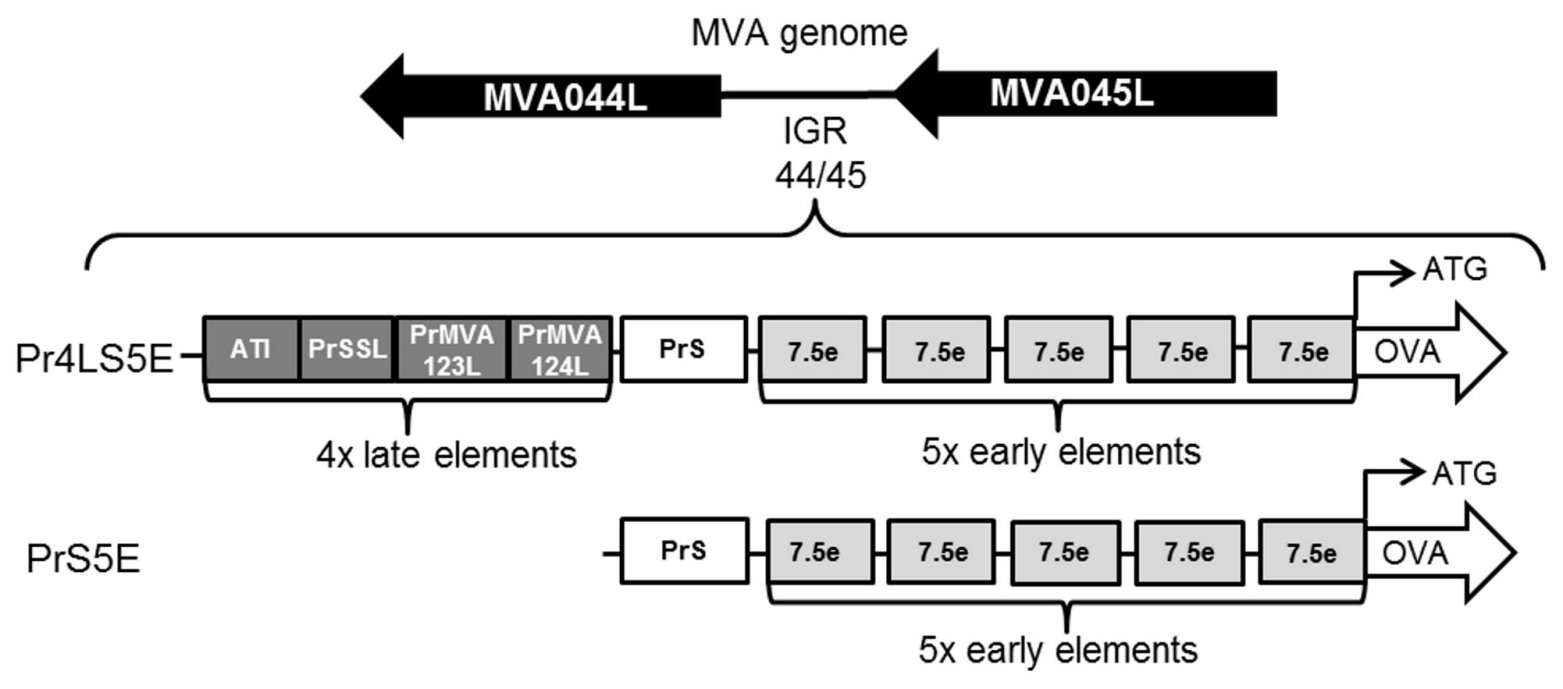
Schematic representation of the novel Pr4LS5E and PrS5E synthetic hybrid promoters. The Pr4LS5E promoter consists of four late elements (dark grey boxes) which include from 5’ to 3’: the ATI late promoter (ATI), the short synthetic late promoter (PrSSL) and the MVA123L and MVA124L late promoters (PrMVA123L and PrMVA124L). The Pr4LS5E contains downstream of these four late elements one PrS promoter sequence (white box) followed by five modified early promoter elements derived from the p7.5k promoter (7.5e; light-grey boxes). The PrS5E promoter consists only of the PrS promoter sequence followed by the five 7.5e elements. The promoters were used to drive the expression of the ovalbumin (OVA) gene. The position of the promoter sequences relative to the start (ATG) of the OVA open reading frame (ORF) is shown. Arrows indicate the direction of transcription. The OVA expression cassettes were introduced into the MVA genome at the intergenic region (IGR) between the MVA genes MVA044L and MVA045L (IGR 44/45) shown as black arrows in the direction of gene transcription.

The synthetic early-late hybrid promoter, pHyb, which contains one late ATI promoter element and five copies of the strong early promoter element derived from the p7.5k promoter, has been shown to induce earlier protein expression and stronger immune responses against an encoded transgene compared to the PrS and p7.5k promoters [23]. In an attempt to improve on this promoter, two novel synthetic promoters, Pr4LS5E and PrS5E, were engineered. The new Pr4LS5E synthetic promoter was engineered to contain four late promoter elements, one PrS sequence, and five p7.5k early elements (Table 1 and Figure 1). The four late promoters in the Pr4LS5E construct included the late ATI promoter, a short strong late promoter element (PrSSL) shown to induce strong late expression in HeLa cells (unpublished data), and the promoters driving the late expression of the MVA123L and MVA124L genes named PrMVA123L and PrMVA124L, respectively (unpublished data). The synthetic PrS5E promoter (Table 1 and Figure 1) contained the compact PrS promoter, which has a strong late element, in place of the ATI late promoter element of pHyb. After the reconstitution and passaging (for at least 5 passages) of the recombinant MVA viruses, the sequences of the respective promoters and insert were checked for DNA sequence integrity. None of the synthetic tandem repeat promoters generated showed sequence alterations.

### Kinetics and strength of expression from novel synthetic hybrid promoters

**Figure 2:**
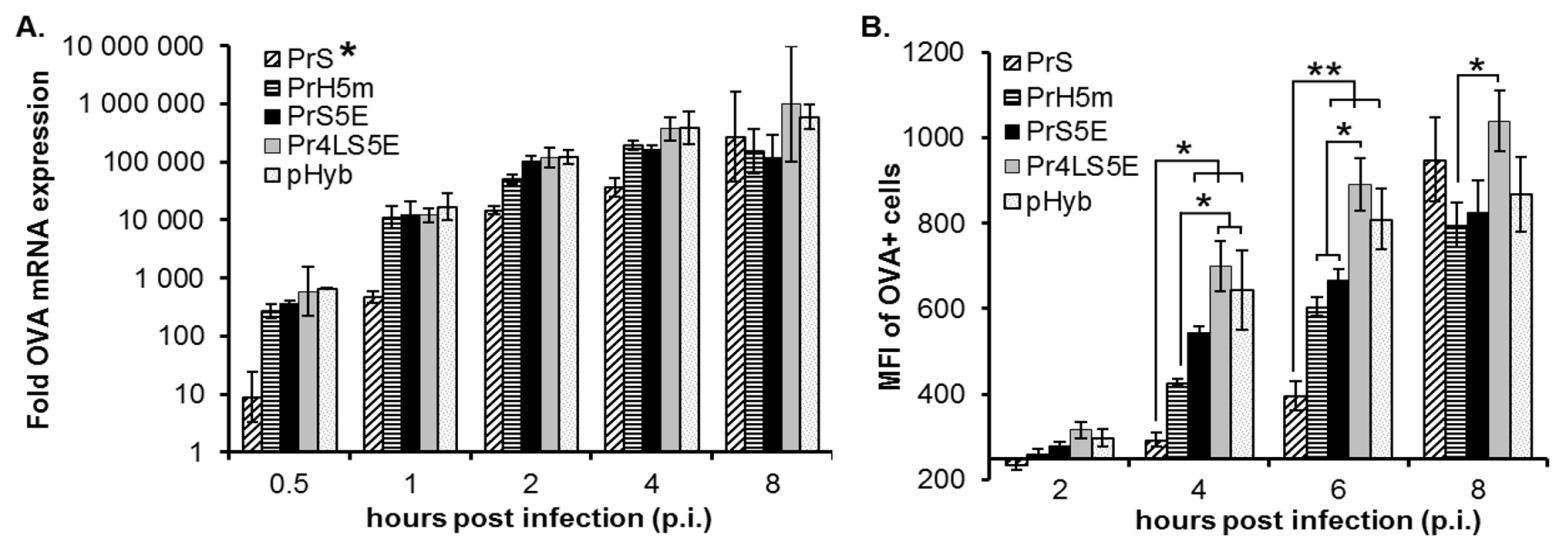
Expression kinetics of novel synthetic hybrid promoters. The kinetics and strength of the indicated promoters driving the expression of ovalbumin (OVA) were analysed in HeLa cells at the mRNA level (A) and at the protein level (B). (A) Samples were collected at 0.5, 1, 2, 4 and 8 hours post infection (p.i.). Total RNA was reverse transcribed and OVA cDNA was detected by qPCR. Fold OVA expression levels were calculated relative to wild-type MVA infected samples after normalization to actin levels using the delta-delta C_T_ method. The average of three independent qPCR runs done in duplicates is shown. Error bars represent ± standard error of the mean (SEM) values. *, indicates the promoter was significantly different (*p* < 0.007) when compared to all other constructs (B) Infected cells were harvested at 2, 4, 6 and 8 hours p.i., stained intra-cellularly for OVA protein and analysed by flow cytometry. Samples staining positive for OVA (OVA+) were gated based on empty vector (MVA-EGFP) infected samples and the median fluorescence intensity (MFI) of OVA+ cells was calculated. For each virus group, three wells were infected using independent virus dilutions. The average MFIs above the background obtained from empty vector infected samples (MFI = 250) are shown. Error bars represent ± SEM values. Statistical differences between the indicated groups are shown by asterisks. * indicates *p* < 0.05; ** indicates p < 0.007.

The kinetics and strength of transcription of the two new synthetic hybrid promoters, PrS5E and Pr4LS5, were compared against three previously characterized poxvirus promoters (PrS, PrH5m and pHyb) using RT-qPCR for the detection of OVA mRNA in infected HeLa cells (Figure 2A). OVA mRNA was detected as early as 0.5 h post infection (p.i.) for all constructs, but significant differences in promoter strength were observed. The PrS5E, Pr4LS5E, PrH5m and pHyb promoters showed the strongest transcriptional activity from 0.5 h up to 4 h p.i. inducing at times up to 10-fold more OVA mRNA when compared to the PrS promoter. However, at 8 h p.i., the PrS promoter increased in strength, resulting in all promoters inducing comparable levels of mRNA at this late time p.i. (Figure 2A). The two novel PrS5E and Pr4LS5E promoters showed similar levels of mRNA expression throughout the time course when compared to the synthetic pHyb promoter and the native PrH5m promoter. This four promoters were overall significantly stronger when compared to the PrS promoter (*p* < 0.007).

In addition to mRNA levels, the relative levels of OVA protein expression were also determined. Figure 2B shows the median fluorescence intensity (MFI) of OVA positive cells for the different viruses above background. The differences observed between the promoters depended on the time point p.i. being compared. At 4 h p.i. the Pr4LS5E and pHyb were the strongest promoters inducing similar levels of protein expression when compared to each other. At this time p.i., both promoters showed stronger expression when compared to the PrS and the PrH5m. At the same time, the PrS5E induced intermediate levels of protein expression which were significantly higher only when compared to the PrS promoter, the promoter inducing the lowest protein levels (Figure 2B). At 6 h p.i., the differences in activity observed between the constructs remained relatively unchanged when compared to the 4 h time point (Figure 2B). However, by 8h p.i. most promoters, including the PrS, had reached similar levels of protein expression with the Pr4LS5E having the highest value but being statistically significant only to the PrH5m (Figure 2B).

Taken together, the weaker transcriptional activity of the PrS promoter at early times p.i. resulted in lower protein levels when compared to the other synthetic hybrid promoters at 4 and 6 h p.i. However, by 8 h p.i. the PrS promoter showed a strong increase in activity which resulted in levels of protein comparable to those observed for all other promoters (Figure 2A and B). The PrS5E and PrH5m promoters, despite inducing approximately equal levels of OVA mRNA when compared to the pHyb and Pr4LS5E (Figure 2A), generated less protein in the infected cells (Figure 2B). The Pr4LS5E and the pHyb showed no significant differences both at the mRNA and protein levels (Figure 2A and B). Thus, the Pr4LS5E promoter can be classified within a category of synthetic early/late hybrid promoters with very strong early expression similar to the previously characterized pHyb promoter.

### Identification of a novel, naturally occurring tandem MVA promoter which drives strong expression of a recombinant antigen

**Figure 3:**
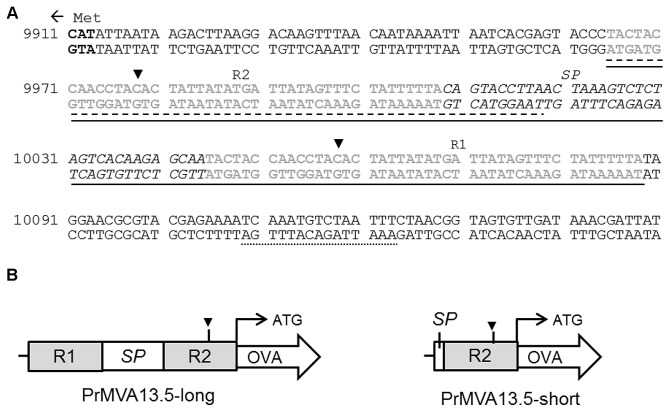
The MVA13.5L promoter. (A) The MVA sequence 250 nucleotides (nt) upstream of the MVA13.5L open reading frame (ORF) with a ‘left’ orientation is shown (GenBank accession number AY603355). The ATG encoding for the methionine (Met) within the MVA13.5L ORF is shown in bold. The arrow indicates the direction of transcription for the MVA13.5L gene. The two transcriptional start sites (TSS) for the MVA13.5L gene are indicated by black arrow heads. TSS1 correspond to nt 9 978 and TSS2 corresponds to nt 10 058 in the MVA genome. The two 44 nt repeats, denoted R1 and R2, are shown as grey sequences. The 36 nt spacer region between R1 and R2, denoted SP, is shown as sequences in italic. The sequences used to construct the PrMVA13.5-long promoter (9 965–10 088 nt) are underlined with a solid line. Sequences used to construct the PrMVA13.5-short promoter (9 965-10 019 nt) are underlined with a dashed line. (B) The features of the native PrMVA13.5-long and PrMVA13.5-short promoter are depicted graphically. The PrMVA13.5-long consists of the two repeated motifs (R1 and R2) shown as grey boxes separated by the spacer region (SP). The PrMVA13.5-short consists of only the R2 motif and 11 nt of the spacer region (SP). The TSS1 is shown as a black arrowhead. The promoters were used to generate an OVA expression cassette introduced into the MVA genome at IGR 44/45. The position of the promoter sequences relative to the start (ATG) of the OVA open reading frame (ORF) is shown. Arrows indicate the direction of transcription.

A 5’ RACE assay was performed on specific cDNAs isolated from MVA infected HeLa cells. The cDNAs targeted for amplification by RACE corresponded to several MVA homologues of VACV genes strongly expressed early during infection [30,31]. Transcriptional start sites (TSS) were then determined and sequences upstream of this site(s) were screened for promoter elements. Using this approach, we identified two different TSS for the MVA13.5L gene: TSS1 at nucleotide (nt) 9978 and TSS2 at nt 10058 (black arrow heads, Figure 3A). The MVA13.5L gene is homologous to the VACV western reserve (WR) 018 gene (VACV-WR018), which is an immediate early (E1.1) gene highly expressed throughout infection [30,31]. Comparison of the sequences 250 nt upstream from the VACV-WR018 and MVA13.5L ORFs showed that these were 100% conserved. A previous report had identified a potential TSS for the VACV-WR018 gene that lies between 9978-9980 nt in the corresponding homologous region in the MVA genome [33], indicating that the TSS1 for the MVA13.5L gene is conserved in MVA and VACV. A detailed analysis of sequences upstream of the MVA13.5L ORF revealed the presence of two 44 nt long identical repeats (R1 and R2) each containing a putative early promoter element (sequences in grey, Figure 3A). R1 and R2 were found almost directly adjacent to each other, separated only by a small spacer region (SP) of 36 nt (sequence in italics, Figure 3A). A promoter sequence for the homologous VACV-WR018 gene has been proposed which corresponds to nt 10109-10123 in the MVA genome (dotted underlined sequence, Figure 3A) [30]. However this predicted promoter does not lie within the R1, R2 or SP regions identified.

To determine the promoter activity of these putative early elements occurring naturally in tandem, two promoter constructs were constructed (Table 1 and Figure 3). The PrMVA13.5-long promoter construct was designed to be comparable to the original viral sequence (solid underlined sequence, Figure 3A) and was composed of R1 and R2 separated by the naturally occurring spacer (Figure 3B). The PrMVA13.5-short (dashed underlined sequence, Figure 3A) was designed as a smaller promoter construct consisting only of R2 and a few nts of the spacer region (Figure 3B). Expression cassettes were constructed containing the OVA gene under the control of the putative PrMVA13.5-long and short promoter constructs and inserted at the IGR44/45 in the MVA genome.

### Kinetics and strength of expression from the two novel MVA13.5L promoter constructs

In order to characterize the activities of the two novel MVA13.5L promoter constructs, these were compared to the Pr4LS5E and PrS5E synthetic hybrid promoters for kinetics and strength of mRNA and protein expression. The Pr4LS5E was chosen for this comparison because it represented one of the promoters with the strongest early activity, similar in kinetics and strength to the pHyb promoter (Figure 2A and B), together with the PrS5E promoter which showed an intermediate phenotype with strong mRNA levels but lower protein levels (Figure 2).

**Figure 4:**
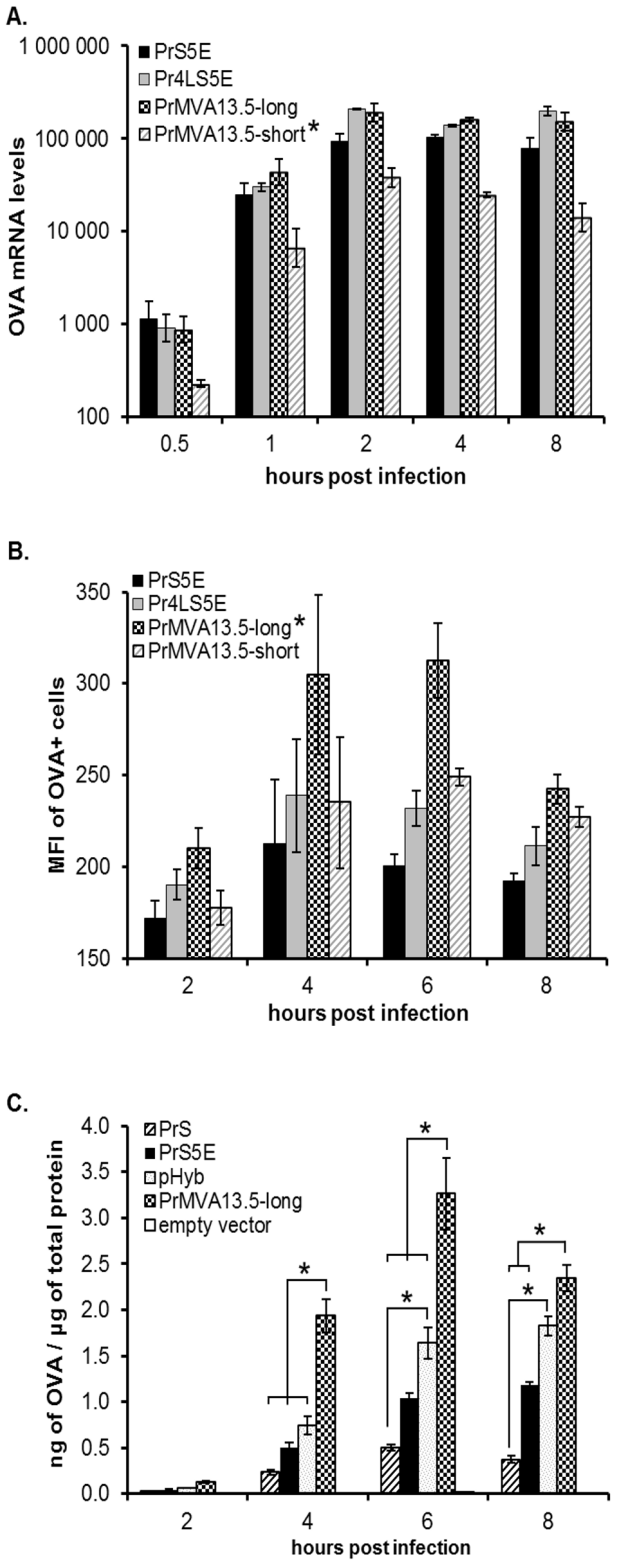
Expression kinetics of the novel MVA13.5L promoter constructs. The kinetics and strength of the PrMVA13.5 promoter constructs were compared to the two novel synthetic hybrid promoters (PrS5E and Pr4LS5E). (A) Cells were collected at 0.5, 1, 2, 4 and 8 hours post infection (p.i.). Total RNA was reverse transcribed and OVA cDNA was detected by qPCR. The fold OVA mRNA levels relative to wildtype MVA were determined using the delta-delta C_T_ method. The average of two independent qPCR runs is shown. Error bars represent ± standard error of the mean (SEM) values. *, indicates the promoter was significantly different when compared to all other promoters (*p* < 0.0002). (B) Infected cells were harvested at 2, 4, 6 and 8 hours p.i., stained intra-cellularly for OVA protein and analysed by flow cytometry. OVA positive (OVA+) cells were gated and the median fluorescence intensity (MFI) of these cells calculated. For each virus group, three wells were infected using independent virus dilutions. The average MFIs of samples with values above the background obtained from empty vector infected samples (MFI = 150) are shown. Error bars represent ± SEM values. *, indicates the promoter was significantly different when compared to all other promoters (*p* < 0.02). (C) Infected MDBK cells were lysed at 2, 4, 6 and 8 hours p.i. All samples were standardized to a total protein concentration of 0.1µg/µL and then used for the determination of OVA protein by ELISA. Values shown for each group are the average of three samples infected with independent virus dilutions, processed independently and assayed in duplicate. Values are reported as nanograms (ng) of OVA per µg of total lysate. Error bars represent ± SEM values. *, denotes a significant difference (*p* < 0.006) between the indicated promoters.

The two MVA13.5L putative native promoter constructs showed detectable transcriptional activity as early as 0.5 h p.i. in HeLa cells (Figure 4A). The early kinetics of mRNA expression observed for these native promoters was similar to that observed for the synthetic PrS5E and Pr4LS5E. Messenger RNA levels for the two native promoters peaked at 2 h p.i. and persisted at similar levels as late as 8 h p.i. (Figure 4A). These results are in agreement with the expression kinetics observed for the VACV-WR018 gene [30,31]. However, despite having similar early kinetics of transcription, the PrMVA13.5-long and PrMVA13.5-short promoters differed in the levels of mRNAs they produced, indicating a difference in transcriptional strength. The PrMVA13.5-long promoter produced similar mRNA levels to those observed for the synthetic promoters PrS5E and Pr4LS5E. In contrast, the PrMVA13.5-short promoter consistently produced mRNA levels which were significantly lower when compared to all other promoters (*p* < 0.0002; Figure 4A).

The activity of the two native MVA13.5L promoters and the two novel synthetic promoters was also compared at the protein level (Figure 4B). Both native MVA13.5L promoters and the two synthetic tandem promoters showed detectable MFI levels above the background as early as 2 h p.i. As expected from the mRNA levels, the PrMVA13.5-long showed higher MFI values compared to the PrMVA13.5-short promoter starting as early as 2 h p.i., but more robustly at 4 and 6 h p.i. When compared to the two synthetic promoters, the PrMVA13.5-long promoter showed higher MFI values particularly at 4 and 6 h p.i. Overall, the PrMVA13.5-long promoter construct showed the highest levels of protein expression compared to all other promoters throughout the time course tested (*p* < 0.02; Figure 4B).

These results demonstrate that the presence of the two 44 nt tandem repeats, each containing an early promoter core element, in the PrMVA13.5-long promoter enhanced transcriptional activity when compared to the PrMVA13.5-short promoter which contained only one of the repeated motifs. Taken together, these data have led to the identification of the PrMVA13.5-long as a new native promoter with very strong early kinetics found as a naturally occurring tandem promoter.

Since MVA is used as a non-replicating live viral vaccine vector, it is important to determine the capacity of this virus to express proteins in cells in which its life cycle is interrupted at an early stage, and in which late protein expression may be compromised. The previous expression analyses were performed in cells of human origin (HeLa) in which MVA replication is inhibited at a late stage of its cycle, after genome replication and late gene expression have occurred [35]. Thus, HeLa cells conveniently allow for the expression of both early and late genes from MVA. However, cell lines from other species such as bovine MDBK cells exhibit an earlier block in virus replication, which results in the inhibition of late protein expression [36]. This could alter the choice of promoters to utilize when designing vaccines for veterinary use.

Since the PrMVA13.5-long promoter elicited very strong early expression, its activity was also characterized in MDBK cells. Three other promoters (pHyb, PrS5E and PrS) were also chosen for this comparison based on the early promoter elements they contained. The PrS promoter was chosen as it contains only one early promoter element, while the pHyb was chosen because it contains five tandem early promoter elements. Finally, the PrS5E was also chosen as it contains the five tandem early promoter elements present in the pHyb and the early element of the PrS promoter.

Figure 4C shows that in the context of a cell line which does not allow for late protein expression, like MDBK cells, the PrMVA13.5-long showed stronger protein expression when compared to promoters with only one early element, such as the PrS. In addition, the PrMVA13.5-long promoter induced higher levels of protein expression when compared to synthetic promoters with tandem early motifs such as the PrS5E and the pHyb (Figure 4C). In particular, the protein expression induced by the PrMVA13.5-long was significantly higher than that observed for all other constructs at 4 and 6 h p.i. At 8 h p.i., the PrMVA13.5-long remained with the highest levels of protein expression; which were significantly higher than those observed for the PrS and PrS5E. Therefore, these results demonstrate that the PrMVA13.5-long promoter is a very strong early promoter capable of inducing high expression levels of a recombinant protein, even in cell lines that restrict the virus replication to an early stage of the infection cycle.

### Cellular immune responses of novel MVA native promoters and synthetic hybrid promoters

Early expression of poxviral genes or transgenes has been correlated with robust CD8 T cell responses specific against the corresponding gene product [23]. To determine the capacity of the two novel hybrid promoters, PrS5E and Pr4LS5E, and the two native MVA13.5L promoters to induce strong transgene-specific T cell responses, C57BL/6 mice were immunized with recombinant MVA viruses expressing OVA under the control of the different promoters.

In clinical trials, a dose of 1x10^8^ TCID_50_ of MVA has been shown to elicit the highest antibody titers with no significant increase in overall reported adverse effects in vaccinated individuals when compared to lower doses [15,37]. Furthermore, the dose of 1x10^8^ TCID_50_ has been found to be optimal in non-human primates (cynomolgus macaques; unpublished data) as well as in mouse models [38]. Therefore, given its relevance in clinical settings and animal studies, a dose of 1x10^8^ TCID_50_ of MVA was used for the evaluation of the immune responses elicited by the different promoters.

The frequencies of CD8 T cells specific for the highly immunodominant H-2K^b^-restricted TSYKFESV *peptide from the VACV B8R protein* were used as a measure of the MVA-specific CD8 T cell responses, while the OVA-specific responses were measured using the SIINFEKL peptide.

**Figure 5:**
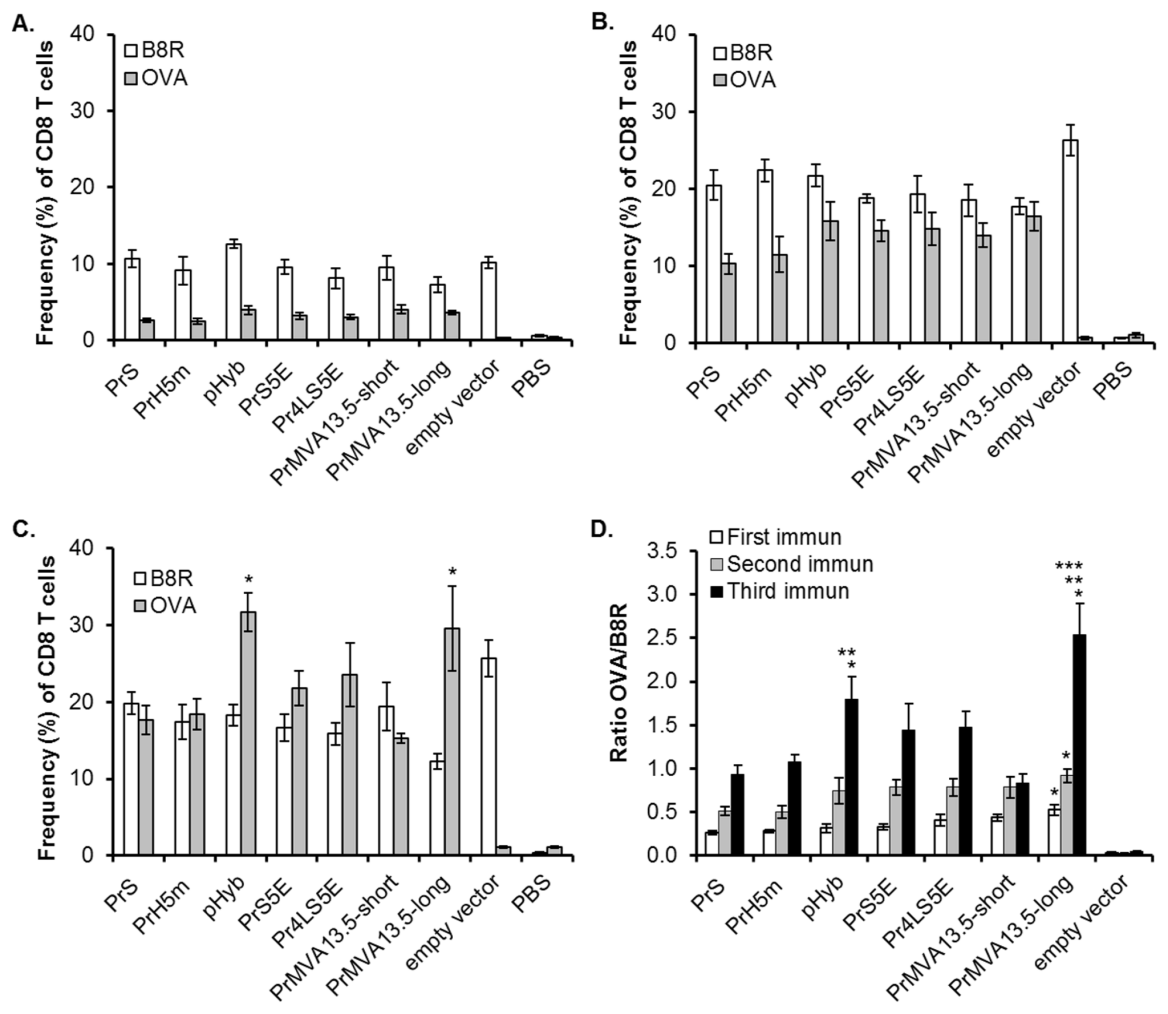
OVA specific CD8 T cell responses. Mice were immunized with different recombinant MVAs expressing ovalbumin (OVA) under the control of the indicated promoters. CD8 T cells from blood were analysed for OVA- and vector- specific responses one week after each immunization by staining for CD8 and CD44 markers along with MHC class I dextramers complexed with the TSYKFESV peptide from the B8R protein or with the SIINFEKL peptide from the OVA protein. CD8 T cells were gated from the lymphocyte population and the frequencies of OVA (gray bars) and B8R (white bars) specific CD8 T cells were obtained as a percentage from the total CD8 T cell population. The frequency of both OVA and B8R specific CD8 T cells were obtained for each mouse and the average for each group calculated. The average frequency of OVA and B8R specific CD8 T cells for each group (+/- SEM) is shown after the first (A), second (B) and third (C) immunizations. *, indicates values with significant difference (*p* < 0.02) when compared to the PrMVA13.5-short, PrS and PrH5m promoters. (D) The frequencies of OVA and B8R specific CD8 T cells were used to calculate OVA- to B8R- specific (OVA/B8R) ratios for individual mice after each immunization (immun). Values reported are the average for each group. Error bars represent +/- SEM. *, indicates values with significant difference (*p* < 0.05) when compared to the PrS; **, indicates values with significant difference when compared to the PrMVA13.5-short; ***, indicates values with significant differences when compared to the PrH5m.

After priming, the CD8 T cell responses against the major vector epitope (B8R) were dominant ranging from 7-13% when compared to the frequency of OVA-specific CD8 T cells, which ranged from 2-4% for the various OVA-expressing constructs (Figure 5A). After a boost immunization, the MVA-specific T cell responses for all constructs increased approximately 2-fold ranging from 18-28% (Figure 5B). The frequencies of OVA-specific T cells were also boosted for all constructs tested. Approximately a 4-fold increase in OVA-specific T cells was observed with percentages ranging from 8-16%. In particular, the PrMVA13.5-long construct showed approximately equal levels of OVA- and vector- specific CD8 T cell responses (16% and 18%, respectively), suggesting that the early strong expression of OVA from this construct was able to induce a potent CD8 T cell response equivalent to the strong immunodominant B8R epitope expressed by the vector backbone after a booster vaccination.

Following a third vaccination (Figure 5C), no major boost in vector-specific responses were observed, with the levels of B8R-specific CD8 T cells remaining approximately constant (12-29%) compared to the levels recorded following the second immunization. In contrast, the OVA-specific CD8 T cell responses were further boosted after the third vaccination (Figure 5C). Significant differences were observed in the percentages of OVA specific CD8 T cells, in particular for the pHyb and the PrMVA13.5-long promoters. These two promoters showed higher levels of OVA specific T cells when compared to the PrMVA13.5-short, PrS and PrH5m promoters (*p* < 0.02; Figure 5C).

In addition, after the third vaccination, the constructs with very strong early expression tended to induce higher frequencies of OVA-specific CD8 T cells than vector-specific CD8 T cells and, by doing so, broke the immunodominance of the vector derived B8R epitope. When the ratios of OVA over B8R frequencies of CD8 T cells were calculated, the new synthetic promoter constructs, PrS5E and Pr4LS5E, showed similar average ratios of 1.4 and 1.5, respectively after the third vaccination (Figure 5D). As previously shown [23], use of the pHyb promoter also resulted in a high OVA-to-B8R average ratio of 1.8 after a third vaccination. From the two native MVA13.5L promoters analysed, the PrMVA13.5-long promoter showed the highest OVA-to-B8R ratio of 2.5 after the third vaccination. The PrMVA13.5-short promoter construct showed similar ratios of OVA-to-B8R ratios compared to the PrS and PrH5m, which were close to 1 (0.8, 0.9 and 1.1, respectively; Figure 5D).

Multiple comparisons of the OVA-to-B8R ratios for all constructs showed that the ratios calculated for the PrMVA13.5-long promoter were significantly higher compared to the PrS after each vaccination (Figure 5D). This was the only promoter tested that showed a significant difference compared to the PrS starting from the first immunization and maintaining this difference after the second and third immunizations. After the third vaccination, the PrMVA13.5-long promoter also showed significantly higher OVA-to-B8R CD8 T cell ratios when compared to the PrMVA13.5-short and PrH5m promoters. By contrast, the pHyb showed a significantly higher ratio of OVA-to-B8R compared to PrS only after the third vaccination. After the third vaccination, the pHyb promoter also showed significantly higher ratios compared to the PrMVA13.5-short promoter construct (Figure 5D). No other significant differences were observed. Taken together, the novel native PrMVA13.5-long promoter was the only promoter tested that was capable of inducing significantly stronger transgene-specific CD8 T cell responses when compared to the PrS after prime and also after subsequent boosts. In addition, these data show that the PrMVA13.5-long construct was superior at inducing OVA-specific responses compared to the shorter promoter construct containing only one repeat motif.

**Figure 6:**
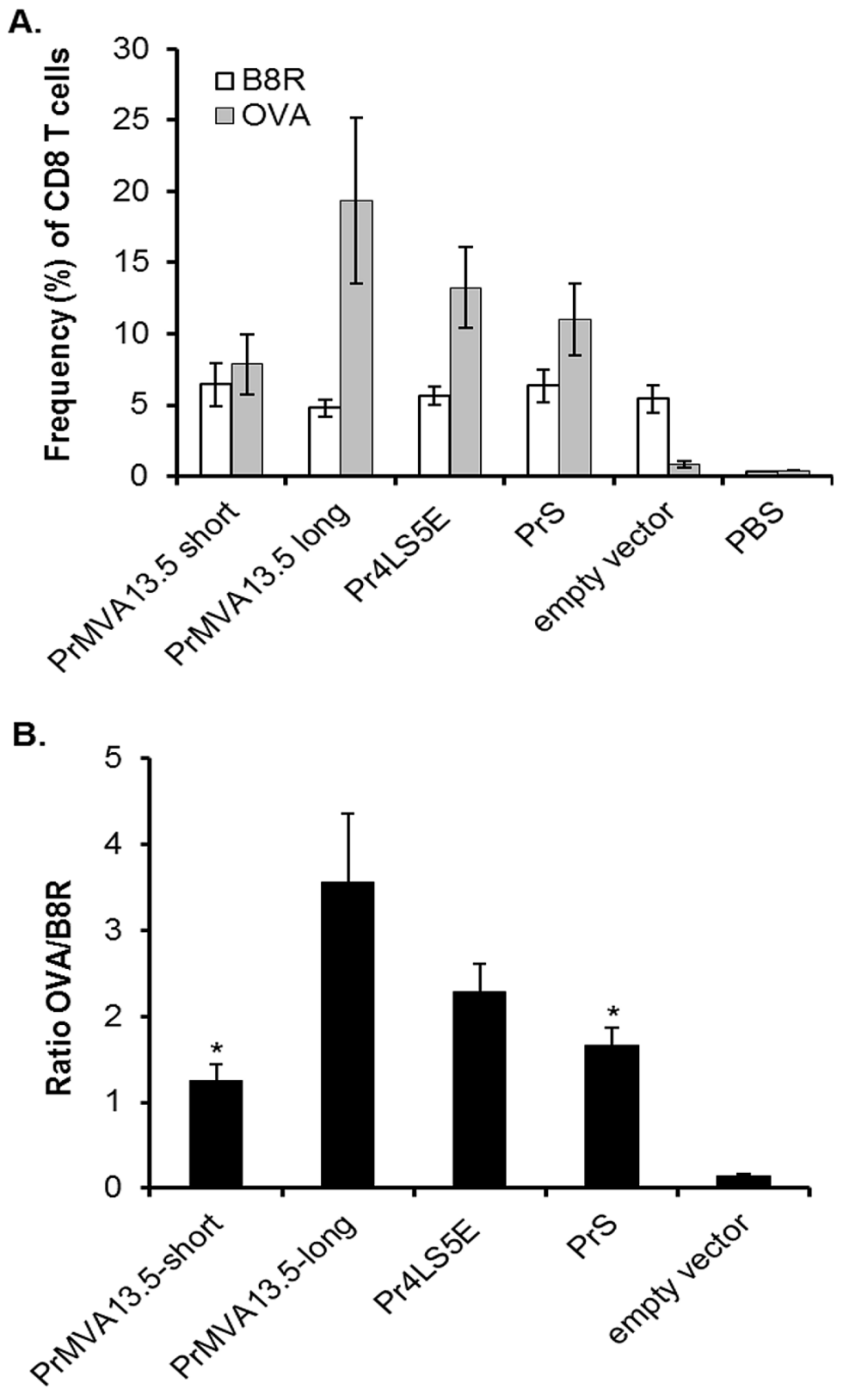
OVA and B8R specific CD8 T cells at the early memory phase. (A) The frequency of OVA- and B8R-specific CD8 T cells were detected for the indicated groups from spleens 10 weeks after the last vaccination using dextramer staining and including memory phenotype markers (CD127 and CD62L). The average for each group is shown +/- SEM values. (B) The average OVA-to-B8R ratios (OVA/B8R) 10 weeks after the third immunization are shown for each group +/- SEM values. Each promoter construct group consisted of 4-5 mice. The empty vector and PBS control groups consisted of 3 mice each. *, indicates the promoter was significantly different when compared to the PrMVA13.5-long promoter (*p* < 0.05).

The frequencies of B8R- and OVA- specific CD8 T cells were also analysed at the early memory phase (10 weeks after the last vaccination) for the PrMVA13.5-long and short native promoter constructs, as well as for the PrS (as a representative promoter with relatively weak early activity) and the Pr4LS5E (as a representative promoter with strong early activity) synthetic promoters (Figure 6A). As expected, the percentages of B8R- and OVA- specific CD8 T cells detected at this time were lower than at the peak of the response. However, the contraction of the OVA specific response was not as strong as that observed for the B8R (Figure 6A). The relative differences in OVA-specific frequencies between the constructs as well as the OVA-to-B8R ratios were maintained at this time (early memory phase) when compared to those one week after the third vaccination (Figure 6A and B). The expression of memory markers (CD127 and CD62L) was also analysed 10 weeks after the third immunization. Approximately, 70-80% of the OVA specific CD8 T cells detected in the blood were of the effector memory phenotype (CD127+, CD62L-) with no significant differences observed between the promoter constructs tested (data not shown). These data demonstrated the presence of a strong OVA-specific memory CD8 T cell population at the start of the memory phase for the constructs tested and in particular for the PrMVA13.5-long promoter, which maintained higher OVA-specific T cell frequencies compared to the PrS, PrMVA13.5-short promoter and the synthetic Pr4LS5E.

### Humoral immune responses of novel MVA native promoters and synthetic hybrid promoters

**Figure 7:**
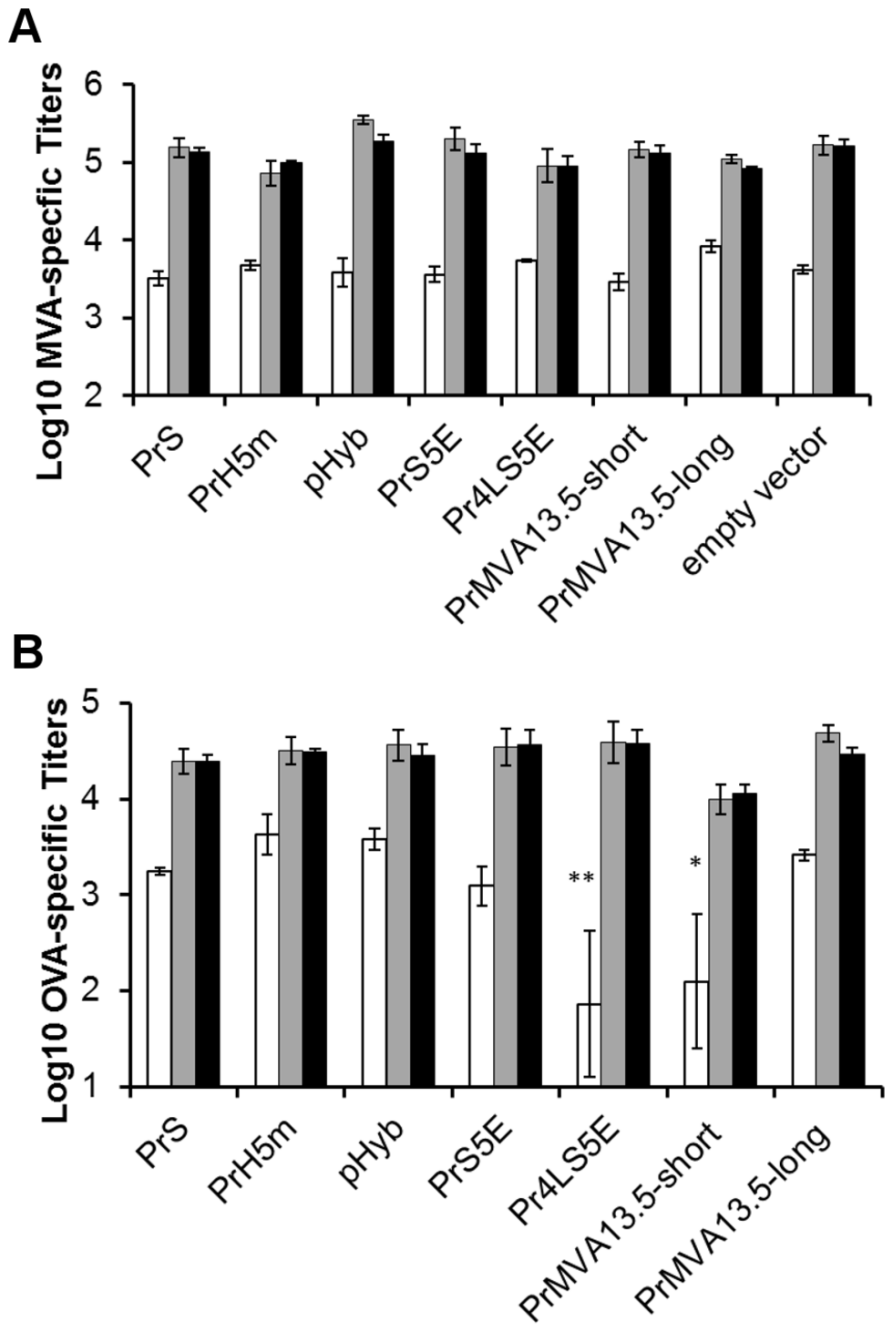
OVA and vector specific antibody titers. Antibody titers specific for MVA (A) or OVA (B) were determined three weeks after the first (white bars), second (gray bars) and third (black bars) immunization (immun). Individual endpoint titers were obtained for each mouse and the respective log_10_ transformed values were calculated. The values reported are the average log_10_ transformed titers for each group +/- SEM values. *, indicates values with significant differences (*p* > 0.05) compared to PrS, PrH5m, pHyb, and PrMVA13.5-long. **, indicates values with significant differences (*p* < 0.05) compared to PrS, PrH5m, pHyb, PrS5E and PrMVA13.5-long.

The humoral responses against the vector and against the OVA protein were also compared between constructs. The average log titers for MVA-specific and OVA-specific antibodies were calculated for each group (Figure 7A and B). All promoter constructs (including the empty vector construct) showed similar MVA-specific antibody titers after the first immunization (Figure 7A). After a second immunization all constructs showed a boost in antibody titers of approximately one log when compared to the first immunization. A third immunization elicited no increase or boost in MVA-specific antibodies suggesting that a saturation level had been reached (Figure 7A). The observation that all constructs elicited similar MVA specific antibody titers after each immunization compared to the wild type (empty vector control) indicated that all mice were properly immunized and that the addition of the OVA transgene had no impact on the vector immunity. All promoter groups tested showed detectable OVA-specific antibodies after the first immunization. These antibody titers were boosted after a second immunization, but not after the third, similar to the effects observed for the MVA-specific titers. This suggests that a third immunization with these constructs provided no advantage for the induction of OVA-specific antibodies, but may still boost the OVA-specific cellular responses. When OVA specific antibody titers were analysed in detail, differences were observed after the first immunization in particular with the Pr4LS5E and the PrMVA13.5-short promoter constructs when compared to the rest of the promoters analysed. For these two groups, seroconversion rates for OVA were 60% and 80%, respectively. All other constructs showed 100% seroconversion with antibody titers above the background for the assay. These lower seroconversion rates after the first vaccination lead to significantly lower average antibody titers for the Pr4LS5E and PrMVA13.5-short promoters when compared to other promoters. A significant difference in OVA-specific antibody titers between promoters after a second and third immunization was not observed.

The observation that strong early promoters such as the PrMVA13.5-long induced similar OVA-specific antibody titers as those observed for the weaker promoters such as the PrS, suggests that induction of antibodies does not correlate with strong early expression of the antigen. From these results, the PrMVA13.5-long promoter was identified as a novel native MVA promoter with the ability to induce strong humoral responses similar in magnitude to those observed for other strong synthetic promoters such as the PrS and pHyb promoters.

## Discussion

In an attempt to identify more potent poxvirus promoters, the MVA genome was searched for novel promoters. Since strong early expression has been shown to correlate with strong CD8 T cell responses from MVA, we focused on identifying promoters for genes with reported strong early and continuous expression during VACV infection and which were also present in MVA. Using this approach, we identified a novel MVA promoter driving the expression of the MVA13.5L gene, a homologue of the immediate early (E1.1) VACVWR-018 gene. This novel native MVA promoter consisted of two 44 nt long repeated motifs (R1 and R2), separated by a small spacer region and containing a highly conserved early promoter sequence motif in both repeats. Analysis of the sequence, using a consensus late promoter motif [28], revealed no obvious late promoter elements. We have also analysed the first 200 nt upstream of all ORFs in the MVA genome and found no other repeated motifs closely spaced together that were greater than 10 nt in length. This observation emphasizes the unique structure of the MVA13.5L promoter. Expression analysis from the PrMVA13.5-short promoter construct showed that one of the repeated motifs found was sufficient for the induction of protein expression and that this sequence was a fully functional immediate early (E1.1) poxvirus promoter. However, the presence of two repeated motifs, as in the native PrMVA13.5-long promoter, resulted in stronger transcriptional activity and superior immune responses. This is the first report, which has identified and characterized a naturally occurring tandem MVA promoter, PrMVA13.5-long, with very strong early kinetics of expression and which is capable of inducing potent immune responses.

In addition to the core promoter sequences, the two MVA13.5L promoter constructs analysed also contained sequences downstream of the transcriptional start sites (TSS) identified for the MVA13.L gene (Figure 3). *S*tudies of early VACV transcription have shown that the early transcription factor (ETF) of VACV makes contacts not only with sequences upstream of the TSS (which correspond to the early promoter elements) but also with sequences downstream of the TSS [39,40]. The contribution of these sequences to the promoter activity of the MVA13.5L promoter constructs remains to be determined.

The MVA13.5L gene encodes for a short 59 amino acid protein of unknown function with approximately 98% homology to the VACV-WR018 protein. Highly conserved homologues of this protein are also found in many other orthopoxviruses, such as ectromelia (EVM014), variola, monkeypox, and cowpox viruses (C10L). The structure of the promoter is also highly conserved. Despite the predicted early expression of the MVA13.5L protein, no major CD8 T cell epitope(s) have been described for it or for its VACV-WR018 homologue in mice, macaques, or humans thus far [41–45]. It is possible that the characteristics of the MVA13.5L protein (cellular processing, binding affinity to MHC molecules and immunogenicity among others) may make it an unfavourable target for CD8 T cells, despite being expressed early during infection and most likely at high levels. Knockout viruses lacking the MVA13.5L would provide information about the function of this protein and whether this gene locus could be used for the insertion of recombinant antigens.

Previous reports have shown that the majority of VACV genes transcribed early during infection (i.e. before 2 h p.i.) and those which are not present in the virion are preferentially recognized by CD8 T cell responses [20]. In this report, the CD8 T cell responses against a recombinant protein were analysed when transcribed from different synthetic promoters containing multiple or single late and early elements. Of the synthetic promoters compared, one contained only one early and one late element (PrS); two contained multiple early elements, but only one late element (pHyb and PrS5E); and a fourth contained both multiple late and early elements (Pr4LS5E). When the CD8 T cell responses, as measured by OVA-to-vector ratios, were compared, we observed that the promoters with multiple optimized early elements (pHyb, Pr4LS5E, and PrS5E) induced stronger responses compared to the PrS containing only one early promoter element. In fact, after three immunizations the pHyb, Pr4LS5E, and PrS5E were able to break the immunodominance of the B8R epitope, while the PrS was not. On the other hand, no obvious advantage was observed between promoters containing one (pHyb and PrS5E) or multiple late promoter elements (Pr4LS5E). When the expression activity of these promoters was compared *in vitro*, the mRNA and protein expression levels of the Pr4LS5E promoter at late time points after infection (8 h p.i.) were slightly higher, but not significantly different, to those observed with the PrS5E and pHyb. This observation suggests that the activity of the multiple late elements in the Pr4LS5E promoter may be compromised perhaps by their positions relative to the open reading frame of the gene and thus may not function optimally. The positions upstream or downstream of the early elements and/or the spacing between the late elements in this hybrid promoter may need to be optimized in order for them to provide a stronger late gene expression. Nevertheless, independent of their late protein expression levels, the two novel PrS5E and Pr4LS5E promoters were identified in this report as having strong immediate early, or E1.1, activity *in vitro* and directed strong cellular immune responses similar to those observed for the pHyb, thus demonstrating their suitability as promoters for use in MVA vectors.

Overall, a strong correlation was observed between strong early transcription and CD8 T cell responses between the promoter constructs analyzed. In MHC-I dextramer binding assays, synthetic and native promoters with very strong early transcription such as the pHyb, Pr4LS5E, PrS5E, and especially PrMVA13.5-long, showed high levels of transgene specific CD8 T cells. In contrast, the PrS promoter that had a relatively weak promoter activity at early times post infection, but comparably strong late expression, induced weaker transgene specific CD8 T cell response even after repeat vaccinations. Thus, in the context of the same antigen, strong early expression (but not late expression) from MVA is an important factor correlating with the induction of strong CD8 T cell responses.

After three vaccinations, the PrS5E, Pr4LS5E, pHyb, and PrMVA13.5-long promoters showed higher levels of OVA- specific CD8 T cells compared to those observed for B8R, a vector derived antigen that is also expressed early during infection [30,31] and is highly immunodominant in VACV infected C57BL/6 mice [41]. The higher OVA-to-B8R ratios observed after repeat vaccinations could be explained by cross-competition of CD8 T cells specific for the two antigens. Reports have shown that CD8 T cell cross-competition regulates the expansion of virus-specific T cells and helps shape the immunodominance hierarchy during boost vaccinations with MVA [46]. In particular, immunodominance after a boost vaccination is correlated with the time of gene expression and thus CD8 T cells specific for early viral antigens are preferentially expanded compared to CD8 T cells specific for late viral antigens [46]. In addition, cross competition during recall responses can also be observed between early viral antigens [46]. In fact, a tendency toward lower B8R frequencies was observed for the promoters with the strongest OVA responses (PrS5E, Pr4LS5E, pHyb, and PrMVA13.5-long), suggesting that OVA-specific T cells for these constructs were preferentially expanded relative to B8R specific T cells. Therefore these results show that the strong early promoters PrS5E, Pr4LS5E, pHyb, and PrMVA13.5-long stimulate potent recall responses after repeat boosts and may provide an advantage in the context of homologous vaccination regimes and immunotherapy.

In clinical trials, MVA is usually administered intradermally (i.d.), intramuscularly (i.m.) or subcutaneously (s.c.). In humans, these three routes generate antibody responses that are dose-dependent with the i.d. route providing potential dose sparing effects [47]. In mice, the levels of antibody and T cell responses against MVA are also dose dependent [38]. In addition, the route of VACV infection in mice has been shown to alter the immunodominance hierarchy of CD8 T cell epitopes with i.p. infection generating T cell responses which are less biased toward immunodominant epitopes when compared to dermal scarification [45,48]. In the present study, the relative strengths of several poxvirus promoters were compared in mice immunized with 1x10^8^ TCID_50_ of MVA administered i.p. It is tempting to speculate that promoters which induce strong T cell responses, such as the PrMVA13.5-long, may generate similar levels of T cell responses at lower doses providing a dose sparing effect especially when administered through a route that can preferentially enhance dominant epitope responses. However, the effects that different doses or routes of administration of MVA would have on the relative strengths of the promoters tested and the immune responses they generate remain to be determined.

Viral genes classified as late, as well as intermediate genes have been shown to be preferred targets for antibody responses during VACV infection [20,21]. In addition, there is also a strong correlation between antigens that are incorporated into the virion (most of which are expressed from late/intermediate genes) and antibody responses [20]. It is still not clear if these correlations are due to the timing of expression during infection (early vs. late), or due to the nature of the antigens (i.e. association with the virion). In this report, the promoters analysed induced both early and late expression of the same antigen (OVA), which is presumed not to be incorporated into the virion. Irrespective of their early activity, all promoters compared showed approximately equal levels of late protein expression (8 h p.i.) and no difference in OVA-specific antibodies was detected irrespective of the type or quantity of late promoter elements used. The use of a strictly late promoter driving the expression of the same antigen has also provided similar antibody titers (data not shown). This suggests that for recombinant antigens expressed from MVA and not incorporated into the virion, the level of early expression does not correlate with antibody responses. The factors that regulate and determine the antibody responses from MVA expressed genes remain elusive.

In summary, we have shown that timely expression of enhanced levels of antigens from MVA vectors can impact immune responses. The use of the best available promoter(s) in the context of a particular poxvirus vector is crucial for the development of a highly effective vaccine or gene therapy vector. However, this can be a challenge particularly for vectors expressing multiple recombinant antigens. The use of the same promoter repeatedly within the same vector may result in genetic instability in which recombination between the promoters can occur [49]. Therefore, the development of polyvalent gene therapy vectors and vaccine candidates expressing multiple recombinant antigens highlights the need for a broader selection of poxvirus promoters which can be used simultaneously without compromising the genetic stability of the vector. The new hybrid promoters and native MVA promoters identified and characterized in this report will enable us to improve future vaccines or gene therapy vectors based on poxvirus vectors. In particular, the strong early expression profile obtained from the new native tandem promoter PrMVA13.5-long in human and bovine cells *in vitro* correlated well with its potent and dominant T cell responses after boost vaccinations in mice. In addition, this promoter was also capable of inducing high antibody titers. Thus, taken together, the ability of the native PrMVA13.5-long tandem promoter to induce strong cellular and humoral responses may improve upon the efficacy of future recombinant vaccines or immunotherapies for human or veterinary use.
